# The Ascent of Artificial Intelligence in Endourology: a Systematic Review Over the Last 2 Decades

**DOI:** 10.1007/s11934-021-01069-3

**Published:** 2021-10-09

**Authors:** B. M. Zeeshan Hameed, Milap Shah, Nithesh Naik, Bhavan Prasad Rai, Hadis Karimi, Patrick Rice, Peter Kronenberg, Bhaskar Somani

**Affiliations:** 1 Department of Urology, Kasturba Medical College Manipal, Manipal Academy of Higher Education, Manipal, Karnataka 576104 India; 2 iTRUE: International Training and Research, Uro-Oncology and Endourology, Manipal, Karnataka India; 3 Department of Mechanical and Manufacturing Engineering, Manipal Institute of Technology, Manipal Academy of Higher Education, Manipal, Karnataka 576104 India; 4 Freeman Hospital, Newcastle upon Tyne, UK; 5 Department of Pharmacy, Manipal College of Pharmaceuticals, Manipal Academy of Higher Education, Manipal, Karnataka 576104 India; 6 Department of Urology, University Hospital Southampton NHS Trust, Southampton, UK; 7 Hospital CUF Descobertas, Lisbon, Portugal

**Keywords:** Machine learning, Artificial intelligence, Endourology, PCNL, Ureteroscopy, ESWL

## Abstract

**Purpose of Review:**

To highlight and review the application of artificial intelligence (AI) in kidney stone disease (KSD) for diagnostics, predicting procedural outcomes, stone passage, and recurrence rates. The systematic review was performed according to the Preferred Reporting Items for Systematic Reviews and Meta-analyses (PRISMA) checklist.

**Recent Findings:**

This review discusses the newer advancements in AI-driven management strategies, which holds great promise to provide an essential step for personalized patient care and improved decision making. AI has been used in all areas of KSD including diagnosis, for predicting treatment suitability and success, basic science, quality of life (QOL), and recurrence of stone disease. However, it is still a research-based tool and is not used universally in clinical practice. This could be due to a lack of data infrastructure needed to train the algorithms, wider applicability in all groups of patients, complexity of its use and cost involved with it.

**Summary:**

The constantly evolving literature and future research should focus more on QOL and the cost of KSD treatment and develop evidence-based AI algorithms that can be used universally, to guide urologists in the management of stone disease.

## Introduction

Artificial intelligence (AI) refers to the computational capability of the machine to mimic and perform human cognitive tasks. Substantial amounts of data are available from the electronic medical records (EMRs) which provide important information, which aids clinicians in shared decision-making and patient counseling [1••]. Machine learning, a subfield of AI, has most readily been applied to clinical research, with techniques including deep learning (DL), artificial neural networks (ANN), natural language processing (NLP), and computer vision being applied across various subfields of urology to aid in the diagnosis as well to predict treatment outcomes [2••].

In the last two decades, there has been a rapid transition in the analysis, treatment, and monitoring of cases with kidney stone disease (KSD). The most recent example being the use of AI in radiomics to identify the stone dimensions from computed tomography (CT) and ultrasound (US) images, detecting stone composition, predicting spontaneous stone passage, and predicting outcomes of endourological procedures. The present systematic review aims to give a comprehensive summary of the contemporary applications of AI in the field of urolithiasis.

### Search Strategy and Article Selection

A review of all English language literature published in the last 2 decades (2000–2020) was conducted in October 2020 using MEDLINE, Scopus, CINAHL, Clinicaltrials.gov, EMBASE, Cochrane library, Google Scholar, and Web of Science. The search strategy was conducted according to the PICO (Patient–Intervention–Comparison–Outcome) [3] criteria where patients with KSD (P) were managed with AI models (I) or traditional biostatistical models (C), and these were examined to evaluate the efficacy of AI models (O). A dedicated search string was then created based on a combination of the following keywords: “Artificial intelligence,” “AI,” “Machine learning,” “ML,” “ANN,” “convolutional networks,” “CNN,” “deep learning,” “DL,” “urolithiasis,” “kidney stone disease,” “ureteric stones,” “nephrolithiasis,” “renal calculi,” “kidney calculi,” and “bladder stones.”

The systematic review was performed according to the Preferred Reporting Items for Systematic Reviews and Meta-analyses (PRISMA) checklist [4]. Only original articles in the English language were included.

Inclusion criteria:

1. Articles on KSD and AI
2. Full-text original articles on all aspect of diagnosis, treatment, and outcomes of stone disease

Exclusion criteria:

1. Editorials, commentaries, abstracts, reviews, or book chapters
2. Animal, laboratory, or cadaveric studies

The literature review was performed according to the inclusion and exclusion criteria. The titles and abstracts were evaluated and after the screening, analysis of the full article text was conducted for selected articles that met the inclusion criteria. The references list of the selected articles was individually and manually reviewed to screen for additional articles of interest. Disagreements about eligibility were resolved by discussion for a consensus decision.

## Results

### Evidence Synthesis

**Fig. 1:**
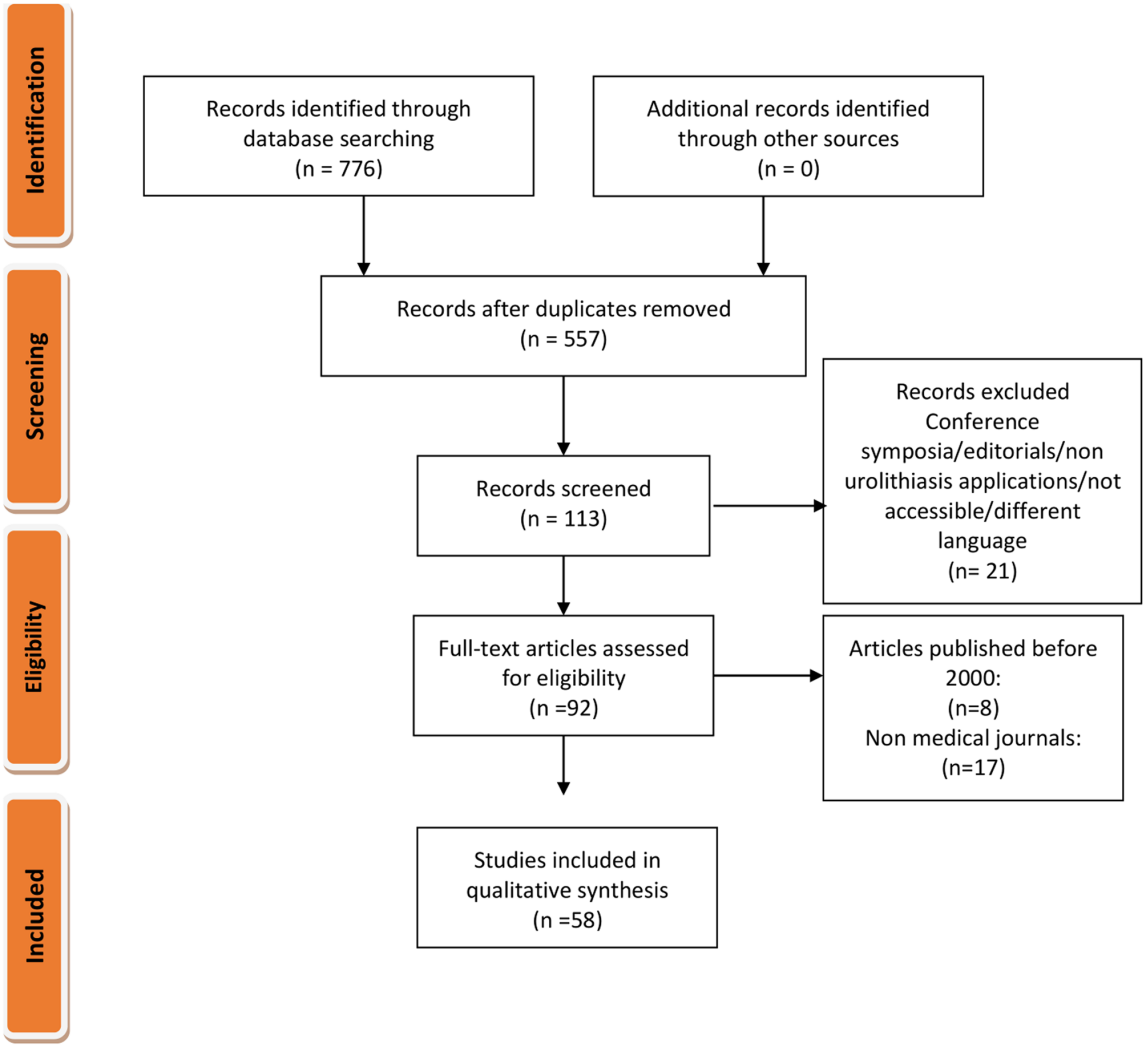
PRISMA flowchart of the literature selection process for articles

**Fig. 2:**
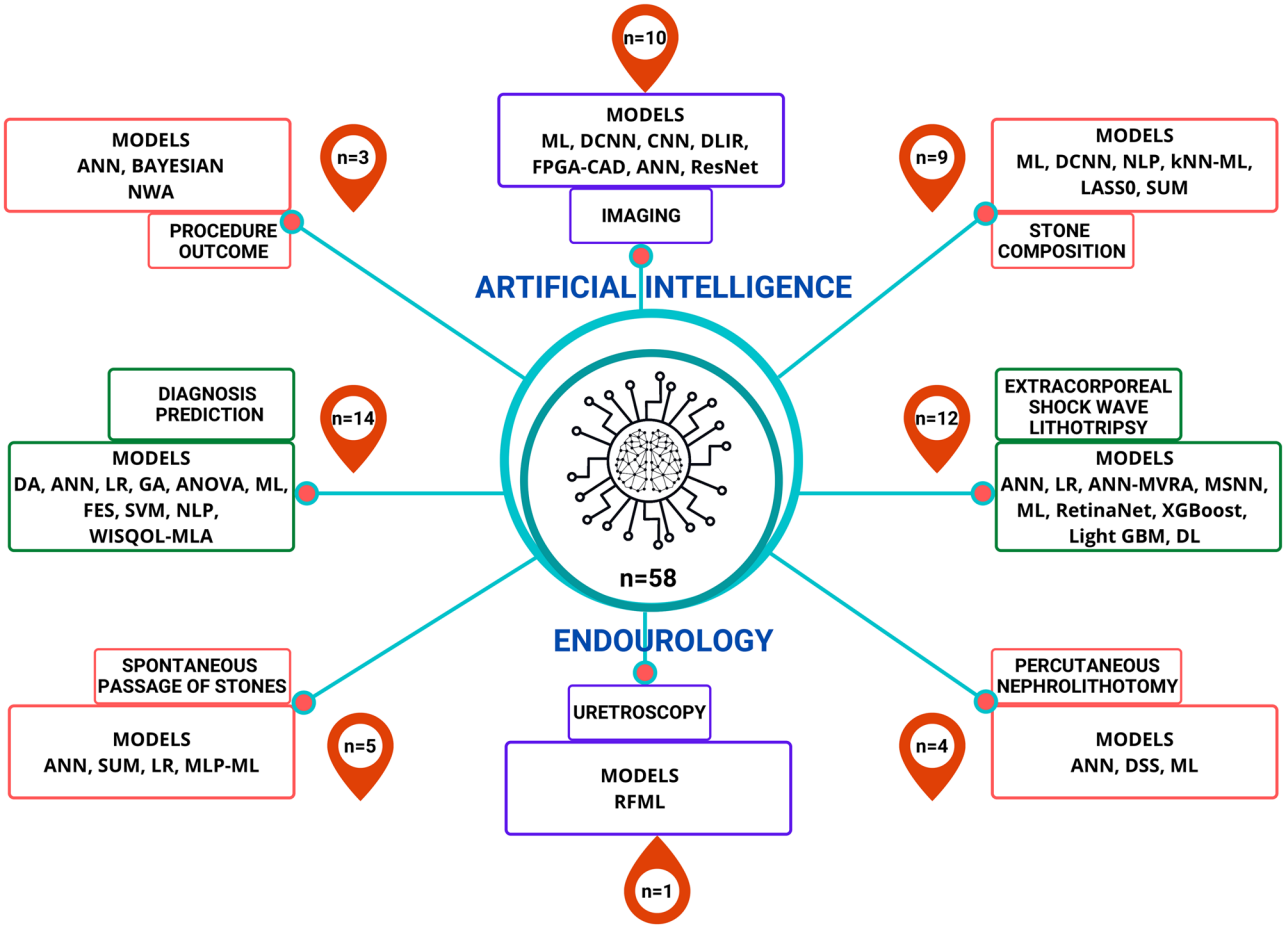
A descriptive summary of number studies on artificial intelligence in endourology and the models used under each field

The initial search identified a total of 557 unique articles. From this list, 113 articles remained following the initial screening, with 92 remaining after a further screening of the abstracts. After additional review of the full-text articles, a total of 58 articles were identified that met our inclusion criteria and were subsequently included in the final review as per PRISMA (Fig. 1). A summary of the included studies is reported in two different tables (Tables 1 and 2) and the AI models used in each study are depicted in Fig. 2.

### Applications of AI

#### Imaging of KSD

Ten studies evaluated the role of AI in KSD imaging for the diagnosis of stone disease. Langkvist et al. [5] used a deep learning convolutional neural network (DCNN) to distinguish ureteric stones from phleboliths based on the thin-slice CT images from the database of 465 patients. The model was tested on 88 scan images. The results showed a sensitivity of 100% with a mean false positive rate of 2.68 per patient [5]. Parakh et al. studied the diagnostic performance of the CNN on CT images for detection of urinary stones in 535 adult patients assumed to have renal calculi using two scanners. The first scanner identified the urinary tract and the second detected the stone. Using nine different variation models, it achieved an accuracy of more than 90%. The study concluded that the efficiency of CNNs can be improved by the use of transfer learning with datasets augmented with labeled images [6].

De Perrot et al. developed an ML model to distinguish kidney stones and phleboliths based on the radiomics feature extraction from low-dose CT (LDCT) images. The model reached an AUC of 0.902, an accuracy of 85.1%, and PPV and NPV of 81.5% and 90.0% respectively [7•]. Jendenber et al. (2020) trained and developed a CNN model to distinguish distal ureteric calculi and phleboliths based on the features of non-contrast CT (NCCT) images and compared these results with assessments reported by seven expert radiologists. The CNN model achieved a significantly higher accuracy of 92%, compared to 86% by the radiologists. The sensitivity, specificity, and AUC of the model to differentiate the distal ureteric calculi and phleboliths were 94%, 90%, and 0.95 respectively [8].

Racine et al. applied novel deep learning image reconstruction (DLIR) methods to check its impact on dose reduction in abdominal CT and compared the results with partial model-based iterative reconstruction (ASiR-V) and filtered back-projection (FBP). In terms of results, DLIR outperformed ASiR-V in all simulated clinical scenarios and at all dose and contrast levels [9].

Krishna et al. proposed a field programmable gate array (FPGA)-based computer-aided detection (CAD) algorithm on US images for detecting abnormality in the kidney, by extracting intensity histogram and Haralick features from the segmented region of interest and trained support vector machine (SVM) and multilayer perception (MLP) classifiers, to classify between renal stones and cyst. The proposed algorithm gave an accuracy of 98.1%, sensitivity of 100%, and specificity of 96.8% in detecting the exact abnormality present on the renal US images. The proposed algorithm and its hardware could help diagnose renal pathology in absence of radiologists and internet connectivity [10].

Li et al. [11] trained a back-propagation ANN to evaluate the best method for localizing renal stone on PCNL between B-mode US and X-ray. Data from 208 patients were used for training while data from 47 patients were used for testing. The results showed that the B-mode US with X-ray was preferred for puncture localization of complex and small renal stones while X-ray was preferred as a single modality in case of simple and larger calculi [11].

Selvarani and Rajendran [12] used the meta-heuristic support vector machine for identifying renal stones on US images. The algorithm was trained with 250 US images (150 with stones and 100 without stones) and achieved an accuracy of 98.8% [12].

Ishioka et al. (2019) used a CNN (ResNet) algorithm for CAD of urinary tract calculi using more than 1000 X-ray KUB images from 3 different hospitals. Eight hundred and twenty-seven images were used as training data and 190 images as test data. In the test dataset, the positive predictive value, sensitivity, and *F*-measure were 0.49, 0.72, and 0.58, respectively [13].

Nithya et al. developed an ANN model for the detection of kidney stones based on the US images using a multi-kernel *k*-means clustering algorithm. The algorithm mainly classified the image as abnormal or normal using the classifier and then the abnormal images were further segmented for the detection of kidney stones. The study showed that the linear and quadratic based model achieved an accuracy of 99.6% [14].

#### Detecting Stone Composition

Nine studies looked at the role of AI in the detection of stone composition. Kreigshauser et al. predicted the stone composition from the CT images by using ML-based algorithms. For stone sizes > 5 mm, they achieved an accuracy of 100% for distinguishing stones containing uric acid (UA) from others. Furthermore, they achieved an accuracy of 75% in distinguishing non-uric acid (non-UA) subtypes [15].

Kazemi and Mirroshandel collected information from 936 patients and derived an ensemble learning model for predicting renal stone composition based on various parameters such as uric acid levels; serum calcium levels; gender; associated symptoms like loin pain, nausea, and vomiting; urinary tract infection; and co-morbidities like hypertension and diabetes. An accuracy of 97.1% was achieved with this model and it showed that these results could be applied in future research activities for predicting stone composition and for recurrence prevention [16•].

Aldoukhi et al. and Black et al. trained a DCNN model to detect stone composition based on the images. Sixty-three stones were taken in the study and the results displayed accuracy of identifying the stone composition of nearly 94%, 90%, 75%, and 86% for uric acid, calcium oxalate, cysteine, and triple phosphate stones respectively. The overall accuracy was 85% in the detection of stone composition. These results have laid a foundation for future research on the detection of the stone composition directly from the endoscopic images and could automate the laser settings for treatment [17, 18•].

Bejan et al. developed StoneX, a natural language processing (NLP) algorithm for mining kidney stone composition in a large-scale electronic health record (EHR) of > 125 million notes. Overall, the system achieved a positive predictive value > 90% for all stone types except for uric acid (PPV = 87.5%). Survival analysis from second stone surgery showed statistically significant differences among stone types (*P* = 0.03). Several phenotype associations were also found such as uric acid—diabetes mellitus type 2; struvite—UTI and neurogenic bladder; hydroxyapatite-neurogenic bladder and pulmonary collapse; and brushite—hypercalcemia or calcium metabolism disorder. This showed that these tools will enable high fidelity kidney stone research from the EHR [19].

Hokamp et al. used dual-energy CT (DECT) images of 200 kidney stones with known composition to train the ML model and predict the main stone composition, in the pure (*n* = 116) and mixed (*n* = 84) kidney stones of sizes 3–18 mm. Both normal-dose and low-dose CT protocols were used for image acquisition. Accuracy was calculated based on stones and voxel both. While the model achieved an accuracy of nearly 90% in predicting the key component of the stone, the lowest accuracy was achieved while detecting the key component of struvite stones [20].

Sacli et al. applied the *k*-nearest neighbor ML algorithm to classify the renal calculi into cystine, calcium oxalate, and struvite stones based on the dielectric properties of the renal calculi. It achieved an accuracy of 98.1% in detecting the stone composition and classifying correctly based on the Cole–Cole parameters [21].

Cui et al. applied the radiomics algorithm to the NCCT images to distinguish between infective and non-infective stones. Twenty-seven radiomic features from CT images were finalized based on the LASSO algorithm. The model was trained with images of clinically confirmed infective (*n* = 98) and non-infective (*n* = 59) patients. The algorithm could differentiate with an accuracy of 90.7%. The sensitivity, specificity, PPV, and NPV were 85.8%, 93.9%, 91%, and 91% respectively [22].

Zhang et al. trained SVM classifiers to assess the accuracy of computed tomography texture analysis (CTTA) in differentiating non-uric acid stones from uric acid stones on NCCT in patients with urinary calculi using commercially available software, with ex vivo Fourier transform infrared spectroscopy (FTIR) as the reference standard. The average SVM accuracy ranged from 88 to 92% (after tenfold cross-validation) with an AUC of 0.965 ± 0.029 with a sensitivity of 94.4% and specificity of 93.7%, thereby concluding that CTTA can be used to accurately differentiate UA stones from non-UA stones in vivo using NCCT images [23].

#### Extracorporeal Shockwave Lithotripsy (ESWL)

Twelve studies looked at the role of AI in ESWL. Poulakis et al. used ANN to predict the outcomes of ESWL used for the treatment of lower calyceal stones using the retrospective dataset of 680 patients, achieving an accuracy of 92%. The predictors of stone clearance included the pattern of dynamic urinary transport, followed by infundibuloureteropelvic angle, body mass index (BMI), caliceal pelvic height, and stone size [24].

Hamid et al. took data of 60 patients in whom ESWL was successfully used to fragments stones and used it to train ANN and subsequently applied it to 22 patients for predicting the number of shockwaves for adequate fragmentation. The overall prediction accuracy was 75% and showed that ANN could identify patients who were not likely to gain any advantage from ESWL and that further studies could improve the prediction accuracy [25•].

Gomha et al. used ANN models to improve the prediction of stone-free status after ESWL for ureteral stones and compared them to a logistic regression (LR) model using a dataset of 984 patients (70% training: 30% test). The sensitivity and specificity of the LR and ANN models were 100%, 0.0%, and 77.9%, 75% respectively with an overall accuracy of 93.2% and 77.7% [26].

Goyal et al. compared the accuracy of ANN and multivariate regression analysis (MVRA) for renal stone fragmentation by ESWL. A total of 276 patients were included, 196 for training the ANN, and 80 for testing it. ANN proved to have a better coefficient of correlation (COC) (power = 0.8343, number of shocks = 0.9329) than MVRA (power = 0.0195, number of shocks = 0.5726), thereby suggesting a better tool to analyze the stone fragmentation by ESWL [27]. Moorthy and Krishnan applied first-order statistical methods and ANN to NCCT images for predicting stone fragmentation using ESWL. The model had accuracy, sensitivity, and specificity of 90%, 80.7%, and 98.4% respectively [28].

Handa et al. developed a method to quantify the hemorrhagic injury to kidneys post ESWL using a Multi-Spectral Neural Network (MSNN) classifier for segmentation and classification of MRI images. The model achieved a high accuracy (79%) and the prediction values correlated very well (*R* = 0.96) with the morphology [29].

Seckiner et al. and Choo et al. used ANN and machine learning methods to accurately predict the outcomes post ESWL for renal calculi and ureteral calculi respectively. Seckiner et al. achieved an accuracy of 88.2%, after ANN trained data of 139 patients and testing it on 32 patients. Choo et al. achieved an accuracy of 92% in their study of 791 patients [30, 31]. Mannil et al. used 5 different AI models and predicted the success rate of ESWL in patients with 5–20 mm kidney stones based on 224 3D-texture analysis features obtained from the CT images. The three features which were found to be significant in predicting the success of ESWL were BMI, skin stone distance, and stone size. The random forest classifier (RF) was found to be the most accurate with an overall AUC of 0.79 [32].

Singla et al. proposed a computer vision algorithm to improve stone targeting during ESWL treatment. The model was trained using a retinanet algorithm on annotated fluoroscopic images of 90 patients and then tested on 12 patients, using a total of 2413 images. The average precision (AP) was 0.7 ± 0.1 while the average detection time (± stdev) was 63 ± 1 ms [33].

Yang et al. used ML methods such as random forest (RF), extreme gradient boosting trees (XGboost), and light gradient boosting method (LightGBM) to predict the success rate of ESWL and also assess the factors affecting the outcomes using a dataset of 358 patients in the ratio of 80:20 as training and test dataset. In predictions for stone-free, LightGBM yielded the best accuracy (87.9%) with AUC 0.84–0.85 and sensitivity and specificity of 0.74–0.78 and 0.92–0.93 respectively [34].

Seltzer et al. applied DL techniques to develop a prediction algorithm to provide better care and improve shared decision making using a dataset from 75/25 randomized split of 46,891 treatments sampled from the International Stone Registry (ISR). The prediction accuracy of stone clearance was 88% with an AUC of 0.95 while predicting complications yielded an accuracy of 77% and an AUC of 0.73 on the validation set [35].

#### Percutaneous Nephrolithotomy (PCNL)

Four studies looked at the role of AI in PCNL. Aminsharifi et al. developed an ANN algorithm to predict outcomes of PCNL by training the machine with data of 200 patients and later applied it on 254 study subjects. The algorithm was able to achieve a sensitivity and accuracy range of 81 to 98.2%. Aminsharifi et al. studied data of 146 adult patients in whom PCNL was done to validate the efficiency of a machine-based learning algorithm for predicting the outcomes after PCNL and to compare results with CROES (Clinical Research Office of Endourological Society) nomogram and Guy’s Stone Score (GSS). This program predicted the PCNL results with an accuracy of up to 95% [36, 37••].

Shabaniyan et al. developed a decision support system (DSS) using ML techniques to predict the outcomes of surgical treatment for renal calculus. The algorithm was trained with a dataset of 254 patients and 26 parameters which comprised variables from patient’s history, stone composition, and laboratory investigations. This model achieved an accuracy of 94.8%, 85.2%, and 95% in predicting outcomes, stent requirement post-procedure, and the need for blood transfusion respectively [38].

Taguchi et al. developed a renal phantom model using automated needle targeting with an X-ray system and compared the feasibility of AI-driven robot-assisted fluoroscopy-guided (RAG) puncture using the US. Seventeen surgeons participated and parameters such as the number of needle punctures, device setup time, fluoroscopic time, and total procedural time were recorded for the analyses. The RAG group was better across all parameters with a statistically significant difference (*p* < 0.001) with a single puncture success rate of 100% in the RAG group [39].

#### Ureteroscopy (URS)

Inadomi et al. developed a Random Forest ML model to predict the requirement of stent insertion post-URS to help improve patient counseling and shared decision making using registry data of 3224 patients who underwent stent insertion. The researchers divided the dataset randomly into training and testing sets at a ratio of 2:1. The variables used were age, prior stent placement, BMI, stone location, procedure acuity, and history of stone surgery The model achieved an AUC of 0.70 on the test set [40].

### Prediction of Outcomes of Endourological Procedures

Alger et al. developed a neural network using pre-and post-procedural data to predict stone-free status for patients treated with ESWL, PCNL, or URS. The model was trained on data from 821 patients and could predict the stone-free rate (SFR) with sensitivity, specificity, PPV, and NPV of 70%, 61%. 61.4, and 72.3 respectively. The model achieved a ROC-AUC of 0.73 [41].

Kadlec et al. designed a model that could predict outcomes of various endourological procedures (PCNL, URS, SWL) and studied the input and outcome variables of 382 renal units. The model predicted stone-free status (defined as stone-free on X-Ray KUB or < 4 mm on CT) with sensitivity and sensitivity of 75.3% and 60.4% respectively. It also predicted the need for a secondary procedure with 98.3% specificity but only 30% sensitivity. This study laid the foundation for the development of similar predictive nomograms in the future [42].

Zhao et al. used Bayesian network meta-analysis (NWA) to assess the efficacy and safety of various minimally invasive procedures for 10–20 mm pediatric renal stones and found that ESWL was inferior to RIRS, mPCNL (mini PCNL), and PCNL for 10–20 mm pediatric renal stones, among which SMP (supermini PCNL) was the most ideal option, associated with the least possibility of complications and highest probability of stone clearance [43].

#### Prediction of Spontaneous Stone Passage (SSP)

Five studies looked at the role of AI in SSP. Cummings et al. designed the ANN model to predict the passage of ureteric calculus based on patient, clinical, and laboratory variables. Out of 181 patients, data from 125 were used to train the model. Of the test dataset of 55 cases, the model correctly predicted SSP in 76% [44].

Parekattil et al. designed and validated a neural network model to predict outcomes and duration of stone passage for ureteral/renal calculi using 6 mm as a cut-off. The model was also evaluated using a 6 mm largest stone dimension cut-off and was tested on 384 patients from 6 different external institutes (other than the design institute). It provided an accuracy of 88% with ROC-AUC 0.9 and duration of passage accuracy of 80% with ROC of 0.8 [45].

Moro et al. applied support vector machines (SVM) to predict the spontaneous passage of ureteric calculi. The machine was trained with a dataset of 1163 patients and the results were compared with those obtained with LR and ANN. The SVM-based approach yielded a sensitivity and specificity of more than 84%. It also suggested the most important factors responsible for SSP in descending order as calculus size, its location, and the duration of symptoms [46].

Kim et al. used LR and MLP-ML models to predict the spontaneous ureteral stone passage using a dataset of 833 patients. AUCs for ROC curves for MLP and logistic regression were 0.859 and 0.847 for stones < 5 mm and 0.881 and 0.817 for stones between 5–10 mm, respectively [47]. Solakhan et al. used the ANN model to estimate the SSP and to determine the effectivity of predictive factors in patients with ureteral stones. A total of 192 patients included a training group (*n* = 132), the validation group (*n* = 30), and a test group (*n* = 30). The accuracy rate achieved was 99.1% in the training group, 89.9% in the validation group, and 87.3% in the test group. It was revealed that certain criteria (stone size, body weight, pain score, erythrocyte sedimentation rate (ESR), and C-reactive protein (CRP)) were relatively more significant for saving treatment cost and time, thereby avoiding unnecessary treatment [48•].

### Various Other AI Applications in Diagnosis and Prediction in Urolithiasis

Chiang et al. predicted the association of stone diseases with genetic polymorphisms as well as dietary, drinking, and exercise habits of the patients using tools like discriminant analysis (DA) and ANN. Four different genes vascular endothelial growth factor, urokinase, cyt-p450c17, and E-cadherin were compared between 151 and 105 patients with and without KSD respectively. Beverages and water consumption and outdoor exercise activities were also considered. The results showed that DA classified 74% and ANN classified 89% correctly. ANN was also proven to be better than DA when all the factors were pooled together [49].

Tanthanuch and Tanthanuch developed an ANN model to identify upper urinary tract calculi prediction using data of 168 patients, divided into 6 categories and 20 variables. The results of testing data showed 100% accuracy with output data between 0–0.38, 0.38–0.65, and 0.65–1 suggestive of being calculi free, probable calculi, and prone to having calculi respectively [50].

Dussol et al. used ANN models to compare 11 clinical and biochemical parameters in 119 males who were idiopathic calcium stone formers and 96 males in the control group. With ANN, supersaturation (ROC = 0.73) and urea (ROC 0.72) were the most discriminants while the other variables such as family history and urinary calcium, citrate, oxalate, urate, sodium, and calcemia, age, and BMI were not statistically different between the two groups. In addition to high supersaturation, the negative impact of protein intake was confirmed [51].

Dussol et al. [52] used ANN models to compare the risk factors (age, BMI, calcemia, calcium oxalate supersaturation, and 24h calciuria, oxaluria, uricosuria, citraturia, urea, and sodium) for idiopathic calcium nephrolithiasis in 119 males and 59 females with and without a family history of renal stones. For men without and with a positive family history, the most discriminant variable was 24h urea (ROC = 0.76) and supersaturation (ROC = 0.67) respectively. For women without and with positive family history, the most significant discriminant was calcemia (ROC = 0.67) and supersaturation (ROC = 0.70) respectively [51].

Eken et al. [53] applied ANN, logistic regression analysis (LR), and genetic algorithm (GA) on data of 227 patients for the diagnosis of renal colic. ANN demonstrated 94.9% and 78.4% sensitivity and specificity respectively. The likelihood ratios were 4.4 (positive) and less than 0.1 (negative). These results can be extrapolated in emergency settings for diagnosis and prediction of colicky pain due to renal calculi and can also help in making clinical decisions [52].

Cauderella et al. applied the ANN model as well as applied conventional statistics (one-way ANOVA and three discriminant analyses: standard, backward stepwise, and forward stepwise) to predict recurrence episodes within 5 years after first clinical diagnosis and metabolic evaluation of real stone based on dataset available from 80 patients with idiopathic calcium stone disease. The model correctly predicted 90% of all cases [54].

Jahantigh et al. developed a fuzzy expert system, as a computer-aided system for the diagnosis of KSD. Results indicated that by examining 21 indicators in the diagnosis of seven cases of kidney disease, KSD was ascertained in 63% and this was compatible with kidney physicians [55]. Chen et al. tested a big data approach to infer and validate a “multi-domain” personalized diagnostic acute care algorithm for KSD combining demographic, clinical, and laboratory variables using statistical and ML models with feature selectors. Data of 38,579 adult patients of which 217 were diagnosed with renal calculi, and 7446 with acute pain (but no renal calculi) were studied. The multi-domain approach using logistic regression yielded an AUROC of 0.86 and a sensitivity/specificity of 0.81/0.82 in cross-validation [56].

Sreelatha and Ezhilarasi also proposed a computer-aided diagnostic tool useful in the automatic classification of kidney images. They divided it into normal, simple cysts, kidney stones, and the less investigated complex cystic renal cell carcinoma (RCC) using SVM classifier and reduced feature set of 18 from the original size of 163 using principal component analysis, achieving an overall accuracy of 96.7% [57].

Li and Elliot assessed the accuracy of NLP in identifying a group of patients positive for ureteric stones on CT KUB reports (*n* = 1874). The accuracy of NLP was 85% with a sensitivity and specificity of 66% and 95% respectively. The low sensitivity and high specificity were due to the lack of feature extraction tools tailored for analyzing radiology text, the incompleteness of the medical lexicon database, and the heterogeneity of unstructured reports [58]. Chen et al. used ML methods to study the risk factors (hypertension, increased protein content in stones, decreased calcium oxalate supersaturation, and old age) causing renal stones > 20 mm using demographic variables, 24-h urine profile, and stone profile data of 277 patients. This model yields sensitivity and specificity of 83% and 56% respectively [59].

Jungmann et al. developed an NLP algorithm that was trained on manual feedback and used to analyze 1714 narrative LDCT reports to automatically capture clinical information and positive hit rates. Urolithiasis was affirmed in 72% of the reports. In 38%, at least one stone was described in the kidney and in 45% at least one stone was described in the ureter. Previous stone history and the combination of obstructive uropathy and loin pain had the highest association with positive urolithiasis (*p* < 0.001) [60]. Luo et al. developed the Wisconsin stone quality of life (WISQOL) machine learning algorithm (WISQOL-MLA) to predict patients QOL based on demographic, symptomatic, and clinical data collected for the validation of WISQOL. The dataset of 3206 patients was split into 70/10/20% training/validation/testing ratio. Gradient boosting obtained a test correlation of 0.622 while DL and multivariate regression obtained a correlation of 0.592 and 0.437 respectively. Quintile stratification on all WISQOL patients obtained an average test AUC of 0.70 for the 5 classes. The model performed best in distinguishing between the lowest (0.79) and highest quintile (0.83) [61]. Kletzmayr et al. used an image-based machine learning approach to screen chemically modified *myo*-inositol hexakisphosphate (IP6) analogues, which enables the identification of a highly active divalent inositol phosphate molecule, which can completely inhibit the crystallization process thereby representing a new treatment option for CaOx nephropathies [62].

### Strengths, Limitations, and Areas of Future Research

The use of a wide variety of AI models and algorithms did not allow us to pool the data together. However, we have included all AI-related endourology articles and summarized its current clinical use and role within endourology.

AI has been used in all areas of KSD including diagnosis, for predicting treatment suitability and success, basic science, QOL, and recurrence of stone disease. However, it is still a research-based tool and is not used universally in clinical practice. This could be due to a lack of data infrastructure needed to train the algorithms, wider applicability in all groups of patients, complexity of its use, and cost involved with it. Future AI studies should also focus more on QOL and the cost of KSD treatment and come up with common algorithms that can be used universally [63••,^64^••].

## Conclusion

The application of AI in KSD and its various subfields appears promising. It is being used for diagnostics, predicting procedural outcomes, stone passage, and recurrence rates. AI-driven management strategies hold great promise for the future and provide an essential step forward in providing more personalized patient care and improving shared decision making. Although not in routine clinical practice currently, we will see a shift in the clinical paradigm as AI applications will find their place in the guidelines and all aspects of KSD management.

**Table 1:** Applications of AI in diagnosis, imaging, and detection of composition of urolithiasis

Applications of AI in imaging and diagnosis of kidney stone disease (KSD)										
Author	Total (n)	Training set	Test Set	Technique/Model	Sensitivity	Specificity	Accuracy	PPV	ROC-AUC	Other Statistical Parameter
Langkvist et al. [5]	465; 437 (28 were removed)	348	88	CNN	100%				0.9971	2.68 false positive per scan
Parakh et al. [6]	535 patients	435	100	CNN			>90%			
De Perrot et al. [7]		369 kidney stones (n=211) phlebolith (n=201)	47 (kidney stones n = 24; phlebolith n=23)	ML classifers: AdaBoostSVMLR Stichaistic gradient descent Gaussian Naïve Bayes kNNRF			AdaBoost: 86.3%SVM:83.2%LR: 82.7% Stochastic gradient descent81.0 % Gaussian Naïve Bayes: 76.6 kNN:71.4 % RF:67.2%	81.50%	0.902	NPV:90%
Jendeberg et al. [8]	Distal ureteric calculi: 267 Phlebolith: 217	Stones: 217 Phlebo liths:167	Stones: 50 Phleboliths: 50	CNN	94%	90%	92%			Semi-quantitative method accuracy: 49%
Krishna et al. [10]	250 normal, 138 stone, and 120 cyst kidney images	SVM: 75 cyst and 75 stone image s	SVM: 45 cyst and 63 stone images	FPGA-based CAD, classifiers: SVM with MLP	100%	96.82%	98.14%			
Li and Elliot [11]	248 (103 PCNL under BUG, 105 X-ray guidance, remaining 40 BUG combined with X-ray)	208	47	Back-propagatio n artificial neural network (ANN); MVR						ANN: R2 = 0.81 MVR: R2 = 0.63
Selvarani and Rajendran [12]	250 US images (150 calculi; 100 healthy)		100 sample US images (50 normal and 50 stone images) using 10- fold approach	PSO-SVMAMM-PSO-SVM			PSO-SVM: 97.4%AMM-PSO-SVM: 98.8%			FAR (%)PSO-SVM 2.6AMM-PSO-SVM 1.8FRR PSO-SVM 3.9AMM-PSO-SVM 3.3
Ishioka et al. [13]	1017	827	190	CNN ResNet	0.72			0.49		F measure:0.58
Nithya et al. [14]	100 (40 normal,30 tumor, 30 stone) for segmentatio n and classification	805	20%	ANN kNNNaïve bias(NB)	ANN: 100%kNN: 66.66% NB: 63.57%	ANN:90% kNN:90% NB:89.7%	ANN: 93.45% kNN: 84.61% NB:83.64% linear + quad ratic based segmentation: 99.61%			
Chiang et al. [49]	151 151 (calcium oxalate stone) patients • 105 healthy			Discrimin ant analysis, ANN			Genetic factorsDA: 64%ANN: 65%• Genetic and Env. Factors DA: 75%ANN: 89%			

Tanthanuch and Tanthanuch [50]	168	100	68	ANN			100% in predicting calculi			
Dussol et al. [51]	119 (stone former)96 (controls)			ANNLDA LRMultivaria te discrimina nt analysis (MVDA)	LDA: 66.4%MVDA: 62.2%	LDA: 87.5%MVDA: 89.6%	LDA: 75.8%MVDA: 74.4%		Univariate DA Supersaturation: 0.73 urinary urea: 0.72family history and urinary calcium: 0.67	
Dussol et al. [52]	178 (stone former)210 (control)			ANN	(Highest) Calcemia: 66.4% in malesUrinary uric acid:62.4% in females	(Highest) Urinary urea:84.4% in malesFamily history:82.5% in females			(Highest) CaOx supersatu ration: 0.731 in males Ur. Calcium in females:0.665	(Highest)predictive powerCaOx supersaturation: 68.3% in males Family history:66.7% in males Family history:66.7 % in females
Eken et al. [53]	227 patients (176 urinary stone, 51 no stones)			ANN genetic algorithm (GA)Logistic regression analysis(LR)	ANN: 94.9% GA: 67.6%LR: 95.5%	ANN: 78%GA: 76% LR: 48%				
Caudarella et al. [54]	80	75%	25%	ANN, LR	ANN: 97.1% LR:51.4%	ANN:82.2%LR:80%	ANN:88.8%LR:67.5%			
Jahantigh et al. [55]	85			Fuzzy expert system						Certainty level of diagnosis: (highest)Kidney stone:0.633 (lowest) Renal tubular acidosis: 0.151
Chen et al. [56]	217 (kidney stones)7446 (pain but no stones)			Diagnostic acute care algorithm kidney stones (DACA-KS)	0.81	0.82			0.86	
Sreelatha and Ezhilarasi [57]	163 features reduced to 18 for analysis			CAD tool			96.90%			
Li and Elliot [58]	1874 CT KUB images			NLP	66%	95%	85%			
Chen et al. [59]	277 patients			ML; LR Superlearner RF LogitBoost Decision tree	(highest)LR: 0.83	(highest)RF: 0.72		(highest)Superlearner: 0.59	(highest)LR: 0.74	(highest)LR: 0.82

Jungmann et al. [60]	1714 images			NLP			Urolithiasis detection: 72%			F1 score for ureteral and renal calculus: 0.90F1 score for ureteral calculus in the findings section:0.76
Luo et al. [61]	3206 patients	70%	20% (test)10% (validation)	DL gradientboosting multivaria te regression (MR)					DL: 0.592Gradient boosting: 0.622MR: 0.4375	
**Applications of AI in detecting stone composition **										
Kazemi and Mirroshandel [16]	936 patients dataset			Bayesian modeDT ANNRule-based classifiers			97.1%(ensemble model)		After pre-processingNB: 99.4Bayesian: 99.4LR: 96.9SVM: 86.9RT: 87.9Ensemble model: 99.6	
Kriegshauser et al. [15]	32 stones dataset (24 stones data of size>5 mm)			ANN SVM DT RF NBT			Distinguish UA and non-UAstones ANN, SVM, and RF (highest): 97% and 100%Distinguish non UA subtypes NBT and RT (highest): 72% and 75%			
Black et al. [18]	63 kidney stones; 17 UA, 21 COM, 7 struvite, 4 cystine, 14 brushite (total of 127 images)			DL-CNNResNet-101	UA 94%COM 90%, struvite 86%, cystine 75%, brushite 71%	UA: 97.83% COM: 97.2%Struvite: 91.84% Cystine: 98.31% Brushite:96.43%				Overall recall: 85%PrecisionUA: 94.12%COM: 95% Struvite: 71.43%Cysteine: 75%Brushite:75%
Bejan et al. [19]	63 kidney stones; 17 UA, 21 COM, 7 struvite, 4 cystine, 14 brushite (total of 127 images)			DL-CNNResNet-101				>90% (COM, CODH,hydro xyapatite, brushite, struvite) uric acid :87.5%		
Hokamp et al. [20]	200 kidney stone(monocrystal line116; dicrystalline 84)	70%	15% (test)15% (validation)	DECTML			Overall (predicting main component): 91.1%			
Sacli et al. [21]	105 calculi(40 patients)			ML,ANNkNN	ANN: 97.1% kNN:98%	ANN: 98.6% kNN:98.6%	ANN: 98.1%kNN: 98.2%			ANNPrecision: 97.2% Recall: 98.6%F1 score: 96% kNN Precision: 97.5% Recall: 98.8%F1 score:98.1%
Cui et al. [22]	135 kidney stones (34 cystine, 34 purines, 32 phosphates, 35 oxalates kidney stones)			ML (kNN and SVM) with Raman spectrosco py	PCA-kNN: 0.963PCA-SVM: 0.963	PCA-kNN: 0.995PCA-SVM: 0.985	PCA-kNN and PCA-SVM: 96.3%			
Zhang et al. [23]	14 patients with 18 UA stones and 31 patients with 32 non-UA stones			SVM Fourier-transform-infrared-spectrosco py (FTIR)	94.40%	93.70%	Differentiating UA from non-UA stones: 88% to 92%		0.965

**Table 2:** Applications of AI in endourological procedures and prediction of outcom

**Applications of AI in ESWL**										
**Author**	**Total (*n*)**	**Training set**	**Test set**	**Technique/model**	**Sensitivity**	**Specificity**	**Accuracy**	**PPV**	**ROC-AUC**	**Other statistical parameter**
Yang et al. [34]	358	286	72	Random Forest	0.74	0.92	-	0.82	0.85	-
				XGBoost	0.75	0.93	-	0.78	0.84	-
				LightGBM	0.78	0.92	-	0.81	0.85	-
Choo et al. [31]	791	791	0	DTA	0.96	0.86	0.92	0.9510	-	-
Mannil et al. [32]	51	34	17	J48	0.71	0.74	-	-	0.72	-
				kNN	0.53	0.68	-	-	0.61	-
				ANN	0.65	0.72	-	-	0.60	-
				RF	0.71	0.74	-	-	0.79	-
				SMO	0.35	0.63	-	-	0.49	-
Seckiner e t al. [30]	203	139	32	ANN	-	-	0.89	-	-	-
Moorth et al. [28]	120	80	40	ANN	0.81	0.98	0.90	-	-	-
Gomha et al. [26]	984	688	296	ANN	0.78	0.75	0.78	-	-	-
Poulakis et al. [24]	701*	101	600	ANN	0.91	0.90	0.92	-	0.9360	-
Singla R et al. [33]	102	90	12	RetinaNet algorithm	-	-	-	-	-	Average precision (AP) = 0.7
Seltzer R et al. [35]	46,891	35,168 (75% of *n*)	11,723 (25% of *n*)	DL	-	-	Success prediction: 0.88 Complicatio ns	-	Success prediction:0.95 Complications prediction:0.73	-
							prediction: 0.77			
Hamid et al. [25]	82	60	22	ANN	-	-	-	-	0.7547	
Goyal NK et al. [27]	276	196	80	ANN	-	-	-	-	-	Power (COC):0.8343 No. of shocks (COC): 0.9329
				MVRA						Power (COC):0.0195 No. of shocks (COC):0.5726
Handa et al. [29]				Multi-spectral neural network (MSNN) classifier	-	-	0.79	-	-	*R* (*n* = 20): 0.9648 *R* (*n* = 10): 0.98
**Applications of AI in PCNL**										
Aminsharifi et al. [37]	454	200	254	ANN, MATLAB software	SFR:0.83	-	82.8	0.83	0.86	-
					Need for PCNL:0.97	-	97.7	0.99	-	
					Need for SWL: 0.98	-	98.2	0.88	-	
					Need for TUL: 0.92	-	92.5	0.92	-	
					Need for DJS: 0.32	-	81.1	0.80	-	
					Need for BT: 0.25	-	85.8	0.85	-	
Aminsharifi et al. [36]	146	-	-	ANN SVM model	-	-	80–95%	-	0.915	-
	254	-	-	QDA	Requirem ent of	SVM classifier is most specific	Overall 94.8% in	-	-	-
				kNN						
Shabaniyan et al. [38]				MLP	Stent placment: 85.2% Requirem ent of blood transfusio n: 95%	across all parameters	predicting outcome of procedure			
				SVM						
**Applications of AI in URSL and prediction of outcomes in endourological procedures**										
Inadomi et al.[40]	3224	2150	1074	RF model	-	-	-	-	0.72	-
Alger et al. [41]	-	821 (310 PCNL, 291 SWL, 120 URS)	-	ANN, NeUROn + + program	70%	61%		61.4	0.73	NPV: 72.3
Kadlec et al. [42]	382 rena l unit s	256	125	ANN, NeUROn + + program	SFR: 75.3% _2_nd procedu re: 30%	SFR: 60.4% 2^nd^ procedure: 98.3%	SFR:69.6% -	SFR: 75.3% _2_nd procedure: 60%	SFR: 0.749 _2_nd procedure: 0.863	SFR-NPV:60.4 2^nd^ procedure: 94.2%
**Applications of AI in prediction of passage of stones**										
Cummings et al. [44]	181	125	55	ANN	-	-	76% (outcome prediction) 100% (stone passage predi ction)	-	-	-
Parekatil et al. [45]	301	141	160	ML, LR	-	-	86.3% (stone passage prediction) 87.3% (duration)	-	-	-
Moro et al. [46]	402	352	50	LR ANN	LR:90.3%	LR: 69.7% ANN: 62.9%	-	-	-	-
				SVM	ANN:94.9% SVM:84.5%	SVM:86.9%				
Kim et al. [47]	833	-	-	MLP LR	-	-	-	-	< 5 mm MLP: 0.859 LR: 0.847 5–10 mm MLP: 0.881 LR: 0.817	-
Solakha et al. [48]	192	132	30 (test) 30 (validati on)	ANN; Alogrithms used were -Quick propagation -Conjugate -Gradient descent, quasi- Newton,—limited memory Quasi- Newton -Online back propagation -Batch-back propagation	-	-	Online back propogation: 99.1% (spontaneous stone passage rate) quick propogation: 92.8%	-	-	-

## References

[1] Beam A, Kohane I (2018). Big data and machine learning in health care. JAMA.

[2] ••Shah M, Naik N, Somani BK, Hameed BMZ. Artificial intelligence (AI) in urology-Current use and future directions: An iTRUE study. Turk J Urol. 2020 Nov;46(Supp. 1):S27-S39. A narrative review about various subsets of artificial intelligence and their application in urology. 10.5152/tud.2020.20117 PMC7731952 32479253

[3] Moher D, Altman DG, Liberati A, Tetzlaff J (2011). PRISMA statement. Epidemiology.

[4] Liberati A, Altman DG, Tetzlaff J (2009). The PRISMA statement for reporting systematic reviews and meta-analyses of studies that evaluate health care interventions: explanation and elaboration. Ann Intern Med.

[5] Langkvist M, Jendeberg J, Thunberg P, Loutfi A, Liden M (2018). Computer-aided detection of ureteral stones in thin-slice computed tomography volumes using Convolutional Neural Networks. Comput Biol Med.

[6] Parakh A, Lee H, Lee JH, Eisner BH, Sahani DV, Do S. Urinary Stone Detection on CT Images Using Deep Convolutional Neural Networks: Evaluation of Model Performance and Generalization. Radiol Artif Intell. 2019 Jul 24;1(4):e180066. 10.1148/ryai.2019180066 PMC8017404 33937795

[7] De Perrot T, Hofmeister J, Burgermeister S (2019). Differentiating kidney stones from phleboliths in unenhanced low-dose computed tomography using radiomics and machine learning. Eur Radiol.

[8] Jendeberg J, Thunberg P, Lidén M (2020). Differentiation of distal ureteral stones and pelvic phleboliths using a convolutional neural network. Urolithiasis.

[9] Racine D, Becce F, Viry A, Monnin P, Thomsen B, Verdun FR, Rotzinger DC (2020). Task-based characterization of a deep learning image reconstruction and comparison with filtered back-projection and a partial model-based iterative reconstruction in abdominal CT: A phantom study. Physica Med.

[10] Krishna KD, Akkala V, Bharath R, Rajalakshmi P, Mohammed AM, Merchant SN, Desai UB (2016). Computer aided abnormality detection for kidney on FPGA based IoT enabled portable ultrasound imaging system. Irbm.

[11] Li G, Liu Z, Zhang Y (2016). Discrimination analysis of B-mode ultrasonography and X-ray on the percutaneous nephrolithotomy localization of urinary stones: a prospective, controlled study. Int J Clin Exp Med.

[12] Selvarani S, Rajendran P (2019). Detection of renal calculi in ultrasound image using meta-heuristic support vector machine. J Med Syst.

[13] Ishioka J, Kobayashi M, Okuno T, et al. Computer-aided diagnosis with a convolutional neural network algorithm for automated detection of urinary tract stones using kub. J Urol. 2019;201(4):e845. 10.1186/s12894-021-00874-9 PMC8340490 34353306

[14] Nithya A, Appathurai A, Venkatadri N, Ramji DR, Anna Palagan C. Kidney disease detection and segmentation using artificial neural network and multi-kernel k-means clustering for ultrasound images. Meas J Int Meas Confed. 2020;149.

[15] Kriegshauser JS, Silva AC, Paden RG (2016). Ex vivo renal stone characterization with single-source dual-energy computed tomography: a multiparametric approach. Acad Radiol.

[16] Kazemi Y, Mirroshandel SA (2018). A novel method for predicting kidney stone type using ensemble learning. Artif Intell Med.

[17] Aldoukhi AH, Law H, Black KM, Roberts WW, Deng J, Ghani KR (2019). Deep learning computer vision algorithm for detecting kidney stone composition: towards an automated future. J Urol.

[18] • Black KM, Law H, Aldoukhi A, Deng J, Ghani KR. Deep learning computer vision algorithm for detecting kidney stone composition. BJU Int. 2020;125(6):920–924. The study using Resnet -101 to automatically detect kidney stones composition using digital photographs of kidney stones. 10.1111/bju.15035 32045113

[19] Bejan CA, Lee DJ, Xu Y, Hsi RS (2019). Performance of a natural language processing method to extract stone composition from the electronic health record. Urology.

[20] Hokamp NG, Lennartz S, Salem J, dos Santos DP, Heidenreich A, Maintz D, Haneder S (2020). Dose independent characterization of renal stones by means of dual energy computed tomography and machine learning: an ex-vivo study. Eur Radiol.

[21] Saçlı B, Aydınalp C, Cansız G, Joof S, Yilmaz T, Çayören M, Önal B, Akduman I. Microwave dielectric property based classification of renal calculi: Application of a kNN algorithm. Comput Biol Med. 2019;112:103366. 10.1016/j.compbiomed.2019.103366 31386972

[22] Cui X, Zhao Z, Zhang G, Chen S, Zhao Y, Lu J (2018). Analysis and classification of kidney stones based on Raman spectroscopy. Biomed Opt Express.

[23] Zhang GMY, Sun H, Shi B, Xu M, Xue HD, Jin ZY (2018). Uric acid versus non-uric acid urinary stones: differentiation with single energy CT texture analysis. Clin Radiol.

[24] Poulakis V, Dahm P, Witzsch U, De Vries R, Remplik J, Becht E (2003). Prediction of lower pole stone clearance after shock wave lithotripsy using an artificial neural network. J Urol.

[25] Hamid A, Dwivedi US, Singh TN (2003). Artificial neural networks in predicting optimum renal stone fragmentation by extracorporeal shock wave lithotripsy: a preliminary study. BJU Int.

[26] Gomha MA, Sheir KZ, Showky S, Abdel-Khalek M, Mokhtar AA, Madbouly K (2004). Can we improve the prediction of stone-free status after extracorporeal shock wave lithotripsy for ureteral stones? A neural network or a statistical model?. J Urol.

[27] Goyal NK, Kumar A, Trivedi S, Dwivedi US, Singh TN, Singh PB (2010). A comparative study of artificial neural network and multivariate regression analysis to analyze optimum renal stone fragmentation by extracorporeal shock wave lithotripsy. Saudi J Kidney Dis Transpl.

[28] Moorthy K, Krishnan M (2016). Prediction of fragmentation of kidney stones: a statistical approach from NCCT images. Can Urol Assoc J.

[29] Handa RK, Territo PR, Blomgren PM, Persohn SA, Lin C, Johnson CD, Jiang L, Connors BA, Hutchins GD (2017). Development of a novel magnetic resonance imaging acquisition and analysis workflow for the quantification of shock wave lithotripsy-induced renal hemorrhagic injury. Urolithiasis.

[30] Seckiner I, Seckiner S, Sen H, Bayrak O, Dogan K, Erturhan S (2017). A neural network - based algorithm for predicting stone - free status after ESWL therapy. Int Braz J Urol.

[31] Choo MS, Uhmn S, Kim JK (2018). A Prediction Model using machine learning algorithm for assessing stone-free status after single session shock wave lithotripsy to treat ureteral stones. J Urol.

[32] Mannil M, von Spiczak J, Hermanns T, Poyet C, Alkadhi H, Fankhauser CD (2018). Three-dimensional texture analysis with machine learning provides incremental predictive information for successful shock wave lithotripsy in patients with kidney stones. J Urol.

[33] Singla R, Lundeen C, Forbes C, Hogarth D, Nguan C. Fluoroscopic targeting of renal calculi during extracorporeal shockwave lithotripsy using a machine learning algorithm. J Urol. 2019;201(4):e474.

[34] Yang SW, Hyon YK, Na HS, Jin L, Lee JG, Park JM, Lee JY, Shin JH, Lim JS, Na YG, Jeon K (2020). Machine learning prediction of stone-free success in patients with urinary stone after treatment of shock wave lithotripsy. BMC Urol.

[35] Seltzer R, Hamilton BD, Klett D, Chen Z, Nakada SY, Gerber G (2019). The prediction of treatment success and complications of shockwave lithotripsy using artificial intelligence. J Endourol.

[36] Aminsharifi A, Irani D, Tayebi S, Jafari Kafash T, Shabanian T, Parsaei H. Predicting the Postoperative Outcome of Percutaneous Nephrolithotomy with Machine Learning System: Software Validation and Comparative Analysis with Guy's Stone Score and the CROES Nomogram. J Endourol. 2020;34(6):692-699. 10.1089/end.2019.0475 31886708

[37] Aminsharifi A, Irani D, Pooyesh S (2017). Artificial neural network system to predict the postoperative outcome of percutaneous nephrolithotomy. J Endourol.

[38] Shabaniyan T, Parsaei H, Aminsharifi A (2019). An artificial intelligence-based clinical decision support system for large kidney stone treatment. Australas Phys Eng Sci Med.

[39] Taguchi K, Hamamoto S, Okada A (2019). Robot-assisted fluoroscopy versus ultrasound-guided renal access for nephrolithotomy: a phantom model benchtop study. J Endourol.

[40] Inadomi M, Ghani K, Kim T, et al. Using a clinical registry and machine learning to predict ureteral stent placement following ureteroscopy. J Urol. 2019;201(4):e460.

[41] Alger PW, Niederberger CS, Turk TMT (2009). Neural network to predict stone free status after SWL, PCNL or ureteroscopy. J Urol.

[42] Kadlec A, Ohlander S, Hotaling J, Hannick J, Niederberger C, Turk TM (2014). Nonlinear logistic regression model for outcomes after endourologic procedures: a novel predictor. Urolithiasis.

[43] Zhao FZ, Li J, Tang L, Li CM, Zhang Y, Wang WY, Chen N, Tian Y. Comparison of efficacy and safety of minimally invasive procedures for 10-20 mm pediatric renal Stones-A bayesian network meta-analysis. J Pediatr Urol. 2020;16(6):771–781. 10.1016/j.jpurol.2020.08.019 32919900

[44] Cummings JM, Boullier JA, Izenberg SD, Kitchens DM, Kothandapani RV (2000). Prediction of spontaneous ureteral calculous passage by an artificial neural network. J Urol.

[45] Parekattil SJ, White MD, Moran ME, Kogan BA (2004). A computer model to predict the outcome and duration of ureteral or renal calculous passage. J Urol.

[46] Dal Moro F, Abate A, Lanckriet GRG (2006). A novel approach for accurate prediction of spontaneous passage of ureteral stones: support vector machines. Kidney Int.

[47] Kim J, Ahn HK, Koo KC, Chung BH, Lee KS. Development of prediction models of spontaneous ureteral stone passage through machine learning: comparison with conventional statistical analysis. J Urol. 2020 Apr;203(Supplement 4):e273. 10.1371/journal.pone.0260517 PMC8635399 34851999

[48] Solakhan M, Seckiner SU, Seckiner I (2020). A neural network-based algorithm for predicting the spontaneous passage of ureteral stones. Urolithiasis.

[49] Chiang D, Chiang HC, Chen WC, Tsai FJ (2003). Prediction of stone disease by discriminant analysis and artificial neural networks in genetic polymorphisms: a new method. BJU Int.

[50] Tanthanuch M, Tanthanuch S (2004). Prediction of upper urinary tract calculi using an artificial neural network. J Med Assoc Thail.

[51] Dussol B, Verdier JM, Le Goff JM, Berthezene P, Berland Y (2006). Artificial neural networks for assessing the risk of urinary calcium stone among men. Urol Res.

[52] Dussol B, Verdier JM, Goff JML, Berthezene P, Berland Y (2007). Artificial neural networks for assessing the risk factors for urinary calcium stones according to gender and family history of stone. Scand J Urol Nephrol.

[53] Eken C, Bilge U, Kartal M, Eray O (2009). Artificial neural network, genetic algorithm, and logistic regression applications for predicting renal colic in emergency settings. Int J Emerg Med.

[54] Caudarella R, Tonello L, Rizzoli E, Vescini F (2011). Predicting five-year recurrence rates of kidney stones: an artificial neural network model. Arch Ital di Urol e Androl.

[55] Jahantigh FF, Malmir B, Avilaq BA (2017). A computer-aided diagnostic system for kidney disease. Kidney Res Clin Pract.

[56] Chen Z, Bird VY, Ruchi R, Segal MS, Bian J, Khan SR, Elie MC, Prosperi M. Development of a personalized diagnostic model for kidney stone disease tailored to acute care by integrating large clinical, demographics and laboratory data: the diagnostic acute care algorithm - kidney stones (DACA-KS). BMC Med Inform Decis Mak. 2018;18(1):72. 10.1186/s12911-018-0652-4 PMC6098647 30119627

[57] Sreelatha P, Ezhilarasi M (2018). Image texture based hybrid diagnostic tool for kidney disease classification. J Med Imaging Heal Informatics.

[58] Li AY, Elliot N (2019). Natural language processing to identify ureteric stones in radiology reports. J Med Imaging Radiat Oncol.

[59] Chen Z, Prosperi M, Bird VG, Bird VY (2019). Analysis of factors associated with large kidney stones: stone composition, comorbid conditions, and 24-h urine parameters-a machine learning-aided approach. SN Compr Clin Med.

[60] Jungmann F, Kämpgen B, Mildenberger P, et al. Towards data-driven medical imaging using natural language processing in patients with suspected urolithiasis. Int J Med Inform. 2020;137:104106. 10.1016/j.ijmedinf.2020.104106 32172185

[61] Luo JW, Nguyen DD, Lim JR, Scotland KB, Bechis SK, Sur RL, Nakada SY, Antonelli JA, Streeper NM, Sivalingam S, Viprakasit DP. Wisconsin quality of life machine learning algorithm for predicting quality of life in kidney stone patients. J Urol 2020;203(Supplement 4):e652

[62] Kletzmayr A, Mulay SR, Motrapu M, Luo Z, Anders HJ, Ivarsson ME, Leroux JC. Inhibitors of Calcium Oxalate Crystallization for the Treatment of Oxalate Nephropathies. Adv Sci (Weinh). 2020;7(8):1903337. 10.1002/advs.201903337 PMC7175250 32328427

[63] New F, Somani BK (2016). A complete world literature review of quality of life in patients with kidney stone disease. Curr Urol Rep.

[64] Geraghty R, Jones P, Herrmann T, Aboumarzouk O, Somani BK (2018). Ureteroscopy seems to be clinically and financially more cost effective than shock wave lithotripsy for stone treatment: systematic review and Meta-analysis. WJU.

